# Is the freezing index a valid outcome to assess freezing of gait during turning in Parkinson’s disease?

**DOI:** 10.3389/fneur.2024.1508800

**Published:** 2025-01-08

**Authors:** Maaike Goris, Pieter Ginis, Clint Hansen, Christian Schlenstedt, Jeffrey M. Hausdorff, Nicholas D’Cruz, Wim Vandenberghe, Walter Maetzler, Alice Nieuwboer, Moran Gilat

**Affiliations:** ^1^Department of Rehabilitation Sciences, Neurorehabilitation Research Group (eNRGy), KU Leuven, Leuven, Belgium; ^2^Department of Neurology, University Hospital Schleswig-Holstein, Christian-Albrechts-University Kiel, Kiel, Germany; ^3^Institute of Interdisciplinary Exercise Science and Sports Medicine, Medical School Hamburg, Hamburg, Germany; ^4^Department of Physical Therapy, Faculty of Medical & Health Sciences and Sagol School of Neuroscience, Tel Aviv University, Tel Aviv, Israel; ^5^Center for the Study of Movement, Cognition, and Mobility, Neurological Institute, Tel Aviv Sourasky Medical Center, Tel Aviv, Israel; ^6^Rush Alzheimer’s Disease Center, Rush University Medical Center and Department of Orthopaedic Surgery, Rush Medical College, Chigaco, IL, United States; ^7^Department of Neurosciences, Laboratory for Parkinson Research, KU Leuven, Leuven, Belgium; ^8^Department of Neurology, University Hospitals Leuven, Leuven, Belgium

**Keywords:** Parkinson’s disease, freezing of gait, wearable sensors, accelerometer, turning, classification, algorithm

## Abstract

**Introduction:**

Freezing of gait (FOG) is a disabling symptom for people with Parkinson’s disease (PwPD). Turning on the spot for one minute in alternating directions (360 turn) while performing a cognitive dual-task (DT) is a fast and sensitive way to provoke FOG. The FOG-index is a widely used wearable sensor-based algorithm to quantify FOG severity during turning. Despite that, the FOG-index’s classification performance and criterion validity is not tested against the gold standard (i.e., video-rated time spent freezing). Therefore, this study aimed to evaluate the FOG-index’s classification performance and criterion validity to assess FOG severity during 360 turn. Additionally, we investigated the FOG-index’s optimal cutoff values to differentiate between PwPD with and without FOG.

**Methods:**

164 PwPD self-reported the presence of FOG on the New Freezing of Gait Questionnaire (NFOGQ) and performed the DT 360 turn in the ON medication state while being videoed and wearing five wearable sensors. Two independent clinical experts rated FOG on video. ROC-AUC values assessed the FOG-index’s classification accuracy against self-reported FOG and expert ratings. Spearman-rho was used to evaluate the correlation between expert and FOG-index ratings of FOG severity.

**Results:**

Twenty-eight patients self-reported FOG, while 104 were classified as a freezer by the experts. The FOG-index had limited classification agreement with the NFOGQ (AUC = 0.60, *p* = 0.115, sensitivity 46.4%, specificity 72.8%) and the experts (AUC = 0.65, *p* < 0.001, sensitivity 68.3%, specificity 61.7%). Only weak correlations were found between the algorithm outputs and expert ratings for FOG severity (rho = 0.13–0.38).

**Conclusion:**

A surprisingly large discrepancy was found between self-reported and expert-rated FOG during the 360 turning task, indicating PwPD do not always notice FOG in daily life. The FOG-index achieved suboptimal classification performance and poor criterion validity to assess FOG severity. Regardless, 360 turning proved a sensitive task to elicit FOG. Further development of the FOG-index is warranted, and long-term follow-up studies are needed to assess the predictive value of the 360 turning task for classifying FOG conversion.

## 1 Introduction

Freezing of Gait (FOG) is one of the most disabling gait problems for many people with Parkinson’s disease (PwPD) and can be defined as “*a brief, episodic absence or marked reduction of forward progression of the feet despite the intention to walk*” (1). FOG expresses itself as two predominant manifestations, namely (i) a full cessation of gait without visible movement, i.e., akinetic FOG; and (ii) a cessation of gait accompanied by alternating trembling motion in the legs usually between 3-8 Hz, i.e., trembling FOG (2). FOG is associated with impaired mobility, a high risk of falls and a reduction in quality of life (3). Up to 80% of PwPD will experience FOG in the advanced stages of the disease (4, 5).

Currently, the treatment of FOG offers limited relief (6). A major impediment for the development of more effective treatments is the difficulty to classify PD freezers from non-freezers and the ability to accurately quantify the severity of FOG (7), due to its episodic and unpredictable nature. Furthermore, the so-called ‘white-coat effect’ causes patients to temporarily experience less FOG when being observed, likely due to heightened arousal and switching to a goal-directed control of locomotion (8, 9). In addition, the typical wide corridors and large uncluttered spaces in the lab or the clinic, in combination with on-state medication screening, often reduces the occurrence of FOG during testing (9).

To classify and assess FOG, self-reported and clinical methods can be used. A widely used self-reported scale is the New Freezing of Gait Questionnaire (NFOGQ) (10), which includes a video of FOG examples presented to the patient. The presence or absence of FOG can be classified using a dichotomous item of this scale, namely “*Did you experience a FOG episode in the past month (yes/no)*?”. Previously, the total score of the NFOGQ showed good agreement with caregivers’ reports of FOG classification (intraclass correlation coefficient [ICC] = 0.78; 95% confidence interval [95%CI] = 0.65–0.87) (10). Importantly, however, the total NFOGQ score is not reliable to use as an outcome of FOG severity in clinical trials given a high test–retest error (11). This variability can be explained by recollection error or by PwPD having diminished insight in their symptoms (11–13). Nevertheless, item 1 of the NFOGQ is still widely used as a screening tool to classify PwPD into freezers and non-freezers (9, 11, 14).

The current gold standard to assess FOG severity is to calculate the percentage time spent with FOG in relation to the total task duration (%TF) by manually annotating video recordings of standardized freezing provoking gait tasks by two independent clinical experts (15). However, annotating FOG from video is highly time consuming, making it an expensive outcome. There are also privacy concerns when videotaping patients (9). In addition, scoring FOG from video is difficult and the clinical experience of the rater is likely to influence the classification and quantification of FOG (16). Consequently, there is growing interest in the development of inertial measurement unit (IMU)-based outcomes for FOG classification and quantification (17). These sensors hold potential for quickly and objectively determining FOG status and measuring its severity (9).

Although FOG is episodic, certain situations are known to frequently elicit FOG, including turning, navigating narrow spaces, and performing a dual-task (1). The most sensitive task to provoke FOG is turning 360-degrees rapidly in both directions for one minute, especially when combined with a cognitive dual-task (18–20). One of the most frequently applied IMU-based algorithms for assessing 360° turning on the spot is the FOG-ratio developed by Morris et al. (21) and further adjusted by Mancini et al. (22). Recently, an update was proposed to enhance the algorithm’s performance to better detect akinetic freezing, the so-called FOG-index (23). The higher the FOG-index, the more FOG-related behavior occurs during the task (21, 22). The FOG-index was shown to be higher for clinically observed (i.e., definite) freezers, compared to self-reported freezers (possible freezers) and clinically confirmed non-freezers (23). The test–retest reliability over a six-week period without an active intervention was excellent for the FOG-index (ICC = 0.897) with a minimal detectable change of 5.04 (23) Furthermore, the FOG-index was shown to reliably identify the number and duration of FOG episodes, irrespective of akinetic or trembling FOG, as compared to patient self-report and clinical ratings. However, this clinical rating was only based on a 0–4 ordinal severity scale (22) and has not yet been validated against gold-standard video-rated %TF. Moreover, no study to date annotated trembling versus akinetic episodes from video to verify that the FOG-index achieved high criterion validity for detecting akinetic FOG. Finally, because non-freezers and healthy older adults can also obtain non-zero values on the FOG-index (17, 23), there is a need to define the most optimal cut-off value to classify PwPD into freezers and non-freezers. Therefore, the aim of the present study was to investigate the classification performance and criterion validity of the FOG-index, as compared to self-reported FOG and gold-standard expert video ratings during the one-minute 360-turning in place task with a dual task.

## 2 Methods

### 2.1 Patients

The PwPD were enrolled through the baseline assessment of the Mobilise-D study (mobilise-d.eu), a large international multicenter trial with the objective to validate digital mobility outcomes (24). A total of 177 PwPD were included: 52 from KU Leuven (Belgium), 90 from Kiel University (Germany) and 35 from Tel Aviv Sourasky Medical Centre (Israel). The assessment occurred between March 2021 and March 2023. Patients were clinically diagnosed with Parkinson’s disease by a neurologist according to the latest diagnostic criteria of the International Movement Disorders Society (25) and had a modified Hoehn and Yahr stage between I-III. Patients with atypical parkinsonian syndromes or known history of stroke were not included (24). All patients gave written informed consent prior to participation in the study, as approved by the Local Medical Ethical Committees of the three centers (Leuven: S64977, Kiel: D 630/20, Tel Aviv: 0551-19-TLV 2020).

### 2.2 Protocol

The assessments took place on a single day and all patients were tested on their usual anti-parkinsonian medications (i.e., practical ON-state). First, as part of the Mobilise-D assessment (24), demographics and clinimetrics were obtained including age, sex, disease duration, the NFOGQ, the Falls Efficacy Scale International (FES-I), the motor section of the Movement Disorder Society Unified Parkinson’s Disease Rating Scale (MDS-UPDRS-III), the postural stability (question 3.12 of MDS-UPDRS-III), the Montreal cognitive assessment (MoCA), the levodopa equivalent daily dose (LEDD) and the current medication status on an ordinal scale (−2 and − 1 = feeling bradykinetic with or without difficulties in daily life activities, 0 = optimal medication status, 1 and 2 = feeling dyskinetic without or with difficulties in daily life activities). Next, patients completed an alternating 360-degrees turning-in-place task (360turn) for one minute with a cognitive dual task, namely the auditory Stroop task (19, 22). Specifically, patients were instructed to turn inside a 40×40 cm taped square on the ground (19) by taking actual steps (i.e., not pivoting on one leg) in alternating directions as quickly and safely as possible for one minute. Patients first started by answering to the Auditory Stroop task while standing, and after a few seconds the assessor gave the starting signal to begin the turning task while continuing the Stroop performance. Patients were allowed to choose the start direction for turning. Five wireless IMU’s (Opals, APDM, Portland, OR, USA) were placed on both feet, both shins, and one at the center of the lower back. In addition, a video camera captured the frontal view of the turning task from neck-to-foot without recording the patients’ faces. An instructor stood beside the patient during task performance for safety purposes. After one minute, the instructor gave the verbal cue ‘stop’ indicating the end of the trial. All data processing occurred post-hoc by video review and analysis of the IMU data.

### 2.3 FOG classification methods

Self-reported classification of FOG status was based on a positive score on item 1 of the NFOGQ (10). Clinically rated classification (serving as the gold standard) was determined by the observation of any FOG episode occurring during video review of the 360 turn by two independent expert raters and verified by a third experienced moderator in case of disagreement. The raters were blinded to the self-reported freezing scores.

For those patients classified as freezers by the experts, the percentage of time spent with FOG relative to the total task duration (%TF) was calculated. This was done by carefully annotating the exact start and stop times of each FOG episode by two raters using the ELAN software (15). Criteria for scoring FOG during turning were obtained from D’Cruz et al. (19). Each FOG episode was also labeled by the experts as predominantly ‘trembling’, characterized by oscillatory movements between 3-8 Hz visible in the legs for more than 50% of the episode, or as predominantly ‘akinesia’, characterized by a lack of progression of the feet without visible trembling in the legs for more than 50% of the episode. Accordingly, patients were grouped into subgroups (overview in Table 1).

**Table 1:** Subgroup overview.

Self-reported freezers	Patients that were classified as a freezer based on a score of 1 on the NFOGQ
Clinically rated freezers	Patients that were classified as a freezer during the one-minute turning task by both experts
Non-freezers	Patients that were not clinically rated, nor self-reported freezers
Unaware freezers	Patients that were clinically rated, but not self-reported freezers
Aware freezers	Patients that were clinically rated and self-reported freezers
Possible freezers	Patients that were self-reported, but not clinically rated freezers

### 2.4 Sensor-based FOG rating

Five wireless IMU’s (Opals, APDM, Portland, OR, United States) were placed on both feet (though these were not used for the final analysis), both shins, and one at the center of the lower back. The IMU’s contained a three-axis accelerometer (6 g) and a three-axis gyroscope (2000dps) recording at 128 Hz. The FOG-index was calculated by dividing the FOG-ratio by the number of completed turns, as described by Mancini et al. (23), in which the FOG-ratio was calculated from the power spectral density (PSD) in the ‘freezing’ band (3-8 Hz) divided by the ‘locomotion’ band (0.5-3 Hz). This was done in the anterior–posterior (AP) or medio-lateral (ML) acceleration signals of the shin IMU’s using the algorithms of Mancini et al. (22), which allowed to obtain the average and maximum value of the Medio-lateral (ML_Mean and ML_Max) and anterior–posterior (AP_Mean and AP_Max) acceleration signals over the entire one minute 360 turn (22). Number of turns were determined based on the gyroscope of the lumbar IMU. A turn was defined as a complete 360° rotation around the longitudinal axis. Partial or incomplete rotations were not considered in the calculations.

### 2.5 Statistical analysis

Statistical analyses were performed using SPSS version 28.0.1.1. The value of 0.05 was set as the significance level. Normality of residual data was calculated with the Shapiro–Wilk test. Only age and MDS-UPDRS-III were normally distributed. Demographical differences between subgroups were calculated using a one-way ANOVA for age and MDS-UPDRS-III, the Kruskal Wallis test for the non-normally distributed data and the Chi-square Test for Independence was used for sex and medication status. Post-hoc tests were used to determine group differences and Bonferroni corrections for multiple comparisons were applied (*p* < 0.008).

To investigate the classification performance and defining a cutoff value of the FOG-index that most accurately classified freezers from non-freezers, a ROC curve was created for each group separately. The cutoff was determined as the point on the ROC curve with the best sensitivity and specificity based on the maximum Kolmogorov–Smirnov metric. Furthermore, area under the curve (AUC) and accuracy rates (AR = [2*AUC]-1) were calculated. For the validation of the FOG-index against expert annotations, Spearman-rho correlation coefficients between sensor metrics and %TF by the experts were used for the expert rated freezers (*n* = 104), and self-reported freezers (*n* = 28) separately. In addition, correlation between expert annotations were calculated for the total %TF (trembling freezing and akinetic freezing episodes), %TF of only the trembling episodes and %TF of only the akinetic episodes.

## 3 Results

### 3.1 Participant characteristics

Data from 164 PwPD were included for the analysis (*n* = 13 were excluded due to technical issues with IMUs and video recordings). We identified 54 non-freezers (32.9%), 28 PwPD with self-reported freezing (17%) and 104 were clinically rated as freezers (63.4%), 82 of whom were unaware (78.8%) and 22 were aware freezers (21.2%). Six self-reported freezers were not observed to freeze during the task by the experts and were classified as possible freezers (21.4%).

Subgroups were similar on a group level for age, MOCA scores, MDS-UPDRS-III, FES-I, medication status and the total number of turns performed in one minute (see Table 2 comparisons between groups). 103 patients felt that they were on their optimal medication status during the test, 28 patients felt more bradykinetic, while only 13 patients felt dyskinetic, the other 20 patients’ medication status was unknown. Sex was not equally distributed between groups with more male freezers than female freezers (Table 2). After correction for multiple comparison, only a significantly different sex distribution was found between the non-freezers and possible freezers.

**Table 2:** Demographics.

Variables	1. No FOG *n* = 54	2. Unaware FOG *n* = 82	3. Aware FOG *n* = 22	Anova/Kruskal Wallis/Chi Square				
	Mean (SD) Min-max	Mean (SD) Min-max	Mean (SD) Min-max	*F*-test	*p*-value	1 vs 2	1 vs 3	2 vs 3
Age. yrs^a^	60.37 (9.4) 37–80	63.90 (9.6) 38–90	63.41 (8.9) 41–75	1.68	0.173	-	-	-
Sex^c^# M F	29 (53.7%) 25 (46.3%)	59 (71.9%) 23 (28.1%)	18 (90%) 2 (10%)	11.50	**0.009**	0.057	0.021	0.05
DD. yrs^b^#	6.81 (4.3) 2–21	7.40 (4.2) 1–21	11.25 (5.4) 4–21	10.85	**0.013**	1.00	0.012	0.029
NFOGQ^b^	0.00 (0.0) 0–0	0.00 (0.0) 0–0	13.45 (6.7) 4–26	161.18	**<0.001**	1.00	**<0.001**	**<0.001**
MOCA^b^	26.30 (2.9) 20–30	26.55 (2.6) 19–30	26.77 (2.6) 22–30	0.64	0.887	-	-	-
FES-I^b^ #	9.45 (3.2) 7–21	9.75 (3.1) 7–17	11.94 (4.0) 7–22	7.37	0.061	-	-	-
MDS-UPDRS-III^a^	25.56 (11.0) 3–48	29.83 (13.7) 4–63	30.00 (13.3) 14–65	1.73	0.163	-	-	-
#Turns^b^	13.89 (6.9) 3–32	11.35 (6.3) 1–50	10.68 (5.6) 3–23	8.33	**0.040**	0.155	0.425	1.00
LEDD^b^	515.40 (264.5) 50–1,098	498.75 (310.9) 0–1,532	753.18 (394.1) 200–1,640	8.84	**0.032**	1.00	0.119	0.020
%TF all^b^	0.00 (0.0) 0–0%	6.37 (10.6) 0.16–60.7%	15.09 (22.1) 0.7–98.9%	121.86	**<0.001**	**<0.001**	**<0.001**	0.655
%TF trembling^b^	0.00 (0.0) 0–0%	1.55 (6.3) 0–43.1%	2.69 (4.6) 0–18.3%	37.25	<**0.001**	**<0.001**	**<0.001**	0.056
%TF akinetic^b^	0.00 (0.0) 0–0%	4.82 (8.2) 0–60.7%	12.40 (22.4) 0.3–98.9%	104.35	**<0.001**	**<0.001**	**<0.001**	0.907

SD = standard deviation; DD = Disease duration; NFOGQ = New Freezing of gait questionnaire; MOCA = Montreal cognitive assessment; FES-I = Falls efficacy scale international; MDS-UPDRS III = Movement Disorders Society Unified Parkinson’s Disease Rating Scale (motor subsection); Turns = total completed full turns during the one-minute turning task; LEDD = Levodopa Doses Equivalency; %TF = percentage time frozen. # Missing data (NO FOG: DD: *N* = 43/54. FES-I: *N* = 49/54. Unaware freezers: DD: *N* = 45/82. FES-I: *N* = 69/82. POSSTAB: *N* = 73/82. Aware freezers: DD: *N* = 16/22; FES-I: *N* = 18/22. Sex *N* = 21/22; Possible FOG: DD=4/6. FES-I=5/6).

a Analyzed with one-way Anova. Bold values indicate significance, group difference are corrected for multiple comparison (*p* < 0.008).

b Analyzed with Kruskal Wallis test. Bold values indicate significance. Group differences are corrected for multiple comparison (*p* < 0.008).

c Analyzed with Chi-square test for independence. Bold values indicate significance. Group differences are corrected for multiple comparison (*p* < 0.008).

The aware freezers showed higher LEDD scores, a longer disease duration compared to the unaware freezers, and a longer disease duration than the non-freezers, but this did not survive correction for multiple comparisons (Table 2). For the %TF, group differences were observed across all FOG episodes, trembling and akinetic episodes. Post-hoc analysis revealed no significant differences between aware and unaware freezers for the %TF. The six possible freezers had similar NFOGQ scores as the aware freezers (10.0 and 13.5, respectively) and were matched on group level for the other demographics and turning characteristics (all *p* > 0.05).

Overall, nine patients experienced only trembling FOG, 62 experienced only akinetic FOG and 33 experienced both. In total, 43 predominantly trembling FOG episodes and 97 predominantly akinetic FOG episodes were annotated.

### 3.2 Criterion validity of FOG classification

**Figure 1:**
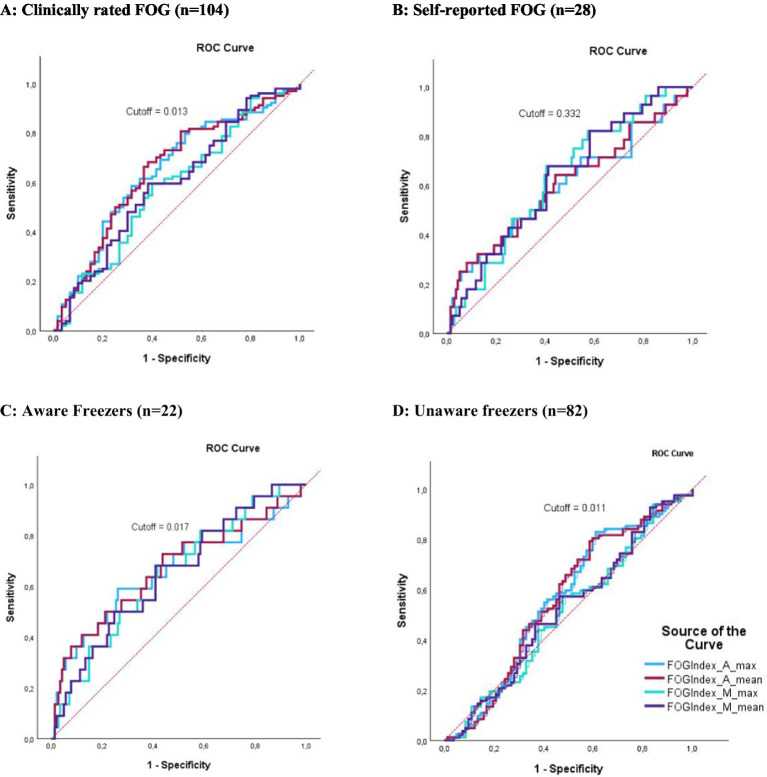
ROC curves representing the classification performance of the FOG-index. The X-axis indicates specificity, and the Y-axis indicates sensitivity. Red dotted lines represent the 0.5 AUC. **(A)** Classification performance of the FOG-index compared to clinically rated FOG. **(B)** Classification performance of the FOG-index compared to self-reported FOG. **(C)** Classification performance of the FOG-index compared to clinically rated FOG combined with self-reported FOG (aware FOG). **(D)** Classification performance of the FOG-index compared to clinically rated FOG without self-reported FOG (unaware FOG). The colors indicate different variations of the FOG index: light blue for AP_Max, violet for AP_Mean, green for ML_Max, and dark blue for ML_Mean.

When compared to the gold-standard expert video annotation, classification metrics (AUC) of the different FOG-index outcomes ranged between 0.51 and 0.65. The ROC curves for the FOG-index are visualized in Figure 1. For the FOG index, AP_Mean provided the best classification with a moderate sensitivity of 68.3% and specificity of 61.7% (cutoff = 0.013, AUC = 0.65, *p* < 0.001, AR = 0.30). For aware freezers (*n* = 22), again AP_Mean provided the best classification with a sensitivity of 72.7% and specificity of 56.3% (cutoff = 0.017, AUC = 0.65, *p* = 0.015, AR = 0.30). For unaware freezers (*n* = 82), the FOG-index could not significantly classify between freezers and non-freezers. Compared to self-reported freezing (*n* = 28), performance of the FOG-index was significant for ML_Mean and ML_Max with an AUC of 0.63 and 0.63 and sensitivity of 69.7 and 67.9% and specificity of 58.8 and 59.6%, respectively. Overall, the cutoff of 0.013 for the FOG-index AP_Mean was the best to distinguish freezers from non-freezers across the clinically rated freezers (*n* = 104). An overview of all cutoff points is presented in Table 3.

**Table 3:** Most optimal cutoff values for classification between freezers and non-freezers.

		Cutoff	Sensitivity (%)	Specificity (%)	AUC	AR	*p*-value
Clinical rated (*n* = 104)	AP_Max	0.021	58.7	68.3	0.65	0.30	0.001*
	**AP_Mean**	**0.013**	**68.3**	**61.7**	**0.65**	**0.30**	0.001*
	ML_Max	0.261	59.6	60.0	0.58	0.16	0.082
	ML_Mean	0.219	59.6	61.7	0.60	0.20	0.041*
Self-reported (*n* = 28)	AP_Max	0.036	46.4	72.8	0.60	0.20	0.115
	AP_Mean	0.093	28.6	91.9	0.60	0.21	0.101
	**ML_Max**	**0.332**	**67.9**	**59.6**	**0.63**	**0.26**	0.016*
	ML_Mean	0.255	67.9	58.8	0.63	0.26	0.018*
Aware freezing (*n* = 22)	AP_Max	0.036	59.1	73.9	0.66	0.32	0.024*
	**AP_Mean**	**0.017**	**72.7**	**56.3**	**0.65**	**0.30**	0.015*
	ML_Max	0.332	68.2	58.5	0.64	0.29	0.019*
	ML_Mean	0.260	68.2	59.2	0.65	0.30	0.015*
Unaware freezing (*n* = 82)	**AP_Max**	**0.011**	**82.9**	**39.0**	**0.56**	**0.12**	**0.165**
	AP_Mean	0.010	79.3	41.5	0.56	0.12	0.170
	ML_Max	0.261	57.3	52.4	0.51	0.01	0.844
	ML_Mean	0.219	57.3	53.7	0.52	0.03	0.675

AUC, Area under the curve. AR, accuracy rates. * Significant at level 0.05. The parameter with the best AUC is indicated in bold.

### 3.3 Criterion validity for assessing FOG severity

Spearman correlations between the FOG index, expert rating of %TF and NFOGQ are presented in Table 4. For the total data set (*n* = 164), many significant, yet weak (all rho <0.4) correlations were found between the FOG-index metrics and the %TF of the expert annotations. When only considering the clinically rated freezers (*n* = 104), all metrics were weakly correlated with %TF for all episodes (rho range = 0.33–0.38; all *p* < 0.05). Correlations with %TF of trembling FOG were relatively smaller (rho = 0.27–0.26, all *p* < 0.05), ML_Max and ML_Mean showed no significant correlation. Weak correlations (rho = 0.23–0.27, all *p* < 0.05) were also found between all FOG-index metrics and akinetic freezing. For the self-reported freezers (*n* = 28), moderate correlations between the FOG-index metrics AP_Max/AP_Mean and %TF of all freezing episodes (rho = 0.40–0.53; *p* < 0.05) and for the akinetic episodes (rho = 0.50; *p* < 0.01) were found, while no significant correlations were found for trembling freezing. None of the algorithm metrics correlated with the NFOGQ total score.

**Table 4:** Spearman rho correlation between FOG-index clinical experts and NFOGQ in the different subgroups.

A: All patients (*n* = 164)	AP_mean	AP_max	ML_mean	ML_max
All FOG (%TF)	**0.37**	**0.37**	**0.28**	**0.25**
Trembling FOG (%TF)	**0.29**	**0.28**	**0.16**	0.14
Akinetic FOG (%TF)	**0.31**	**0.3**	**0.25**	**0.22**
NFOGQ	0.13	0.13	0.17	**0.17**

B: Clinical rated FOG (*n* = 104)	AP_mean	AP_max	ML_mean	ML_max
All FOG (%TF)	**0.38**	**0.37**	**0.34**	**0.33**
Trembling FOG (%TF)	**0.27**	**0.26**	0.14	0.13
Akinetic FOG (%TF)	**0.24**	**0.23**	**0.27**	**0.24**
NFOGQ	0.17	0.17	0.18	0.17

C: Self-reported FOG (*n* = 28)	AP_mean	AP_max	ML_mean	ML_max
All FOG (%TF)	**0.53**	**0.51**	**0.4**	0.32
Trembling FOG (%TF)	0.21	0.19	0.05	−0.02
Akinetic FOG (%TF)	**0.5**	**0.5**	0.35	0.27
NFOGQ	−0.08	−0.07	0.07	0.09

Bold values indicate significance (*p* < 0.05).

## 4 Discussion

The aim of this study was: (I) to investigate the performance of the previously developed and commonly used FOG-index to classify PwPD into freezers and non-freezers; (II) to determine the best cutoff value for classification; and (III) to determine criterion validity of the algorithm to assess FOG severity as compared to gold-standard video annotations and this for all FOG, as well as for trembling versus akinetic episodes. This is the first study to investigate the performance of the FOG-index during the most sensitive FOG-provoking DT 360 turn in a large cohort of PwPD (*n* = 164) ON medication using expert ratings as the reference test.

### 4.1 Classification performance

Somewhat surprisingly, we found that a large proportion of patients clinically rated as freezers were unaware of their freezing. Only 28 PwPD reported freezing on the NFOGQ while 104 were classified as freezers by the experts during the 360 turn. This may be explained by our result that unaware freezers tended to have a shorter disease duration than aware freezers and may not have experienced troublesome FOG yet in daily life. Indeed, people rarely perform 360° turns with a DT in daily life, while this is known to be the most sensitive task to elicit FOG (19, 20). Our results suggest that this task might therefore be able to pick up freezing early, even before patients become aware of it. This observation should next be verified by future long-term follow-up work to determine if the turning task can predict conversion to ‘aware’ FOG. In the aware freezing group, the FOG-index had a sensitivity of 72.7% and specificity of 56.3%, while in the unaware group the sensitivity and specificity were 82.9 and 39%, respectively. The results suggest a trend wherein the aware group had a higher %TF for trembling freezing compared to the unaware group. Although statistical significance was not reached (*p* = 0.056), this finding suggests that the algorithm was better at picking up more severe freezers. An assigned classification as a non-freezer by the FOG-index in the unaware group must be interpreted with caution, since there was a 61% chance that the patient was in fact a freezer. However, when the algorithm classified an individual as a freezer, there was a 82.9% certainty that the person indeed was a freezer. It can be concluded that the FOG-index is able to pick up aware freezers, but that it has low specificity to pick up the less severely affected unaware freezers, whom a clinical expert is able to identify using the DT turning task. Therefore, awareness of the FOG-index’s limitations is crucial when using it for FOG classification, as it may not reliably classify all individuals, especially those with milder symptoms.

The FOG-index AP_Mean is the best classifier to distinguish freezers from non-freezers compared to expert ratings with a sensitivity of 68.3% and specificity of 61.7%. This is in line with previous studies where the anterior–posterior acceleration was also found to be the best classifier for freezing (22). The accuracy of the FOG classification found in this study is relatively low compared to previous studies, where sensitivity of IMU algorithms ranged between 63–100% (26). However, measurements in these studies were most often taken in the OFF-medication state when FOG severity is known to be higher, but this is often not the scenario in clinical settings nor daily life (27).

In this study there were 6 possible freezers, who did not show freezing during the DT turning task. An explanation for these results is the so-called white-coat effect whereby PwPD exert heightened attention during testing when being observed which reduces the chances of FOG (9). Another possible explanation is that these patients experience FOG in their daily life when medication wears off, and that the turning task would have elicited FOG if testing was done in OFF (20, 28).

### 4.2 Criterion validity for FOG severity

Only moderate correlations (all rho≤0.53) were found between %TF and any of the FOG metrics from the FOG index, which is in line with the findings from D’Cruz et al. (19). Previous studies have highlighted challenges in detecting akinetic freezing using the FOG-ratio, because a lack of visible trembling movement in the legs inherently provides little features on IMU data to detect such episodes (9, 17, 29). Moreover, the widely used FOG-ratio in particular regards any movement in the <3 Hz range as ‘normal’ and so could miss akinetic FOG (17). To improve algorithm performance for akinetic freezing the FOG-index was developed (23). Our results showed significant, yet weak correlations between the FOG-index and %TF for akinetic FOG, indicating that the FOG-index is indeed capable at picking up akinetic FOG, but that the criterion validity is still limited. Even though trembling FOG is typically considered the most prevalent manifestation (2, 12), in this study we observed 97 akinetic episodes and only 43 trembling episodes during this challenging DT 360 turn. Possibly, the combined demand for motor asymmetry and balance requirements of turning with a dual-task could lead to more frequent akinesia (30, 31), potentially leading to greater fear of falling in these patients (32, 33). External validation studies using a greater variety of FOG provoking tasks in a cohort with more trembling freezing are needed to support this interpretation.

Contrary to previous literature, we did not find a correlation between the FOG-index and the NFOGQ (22, 23). Previous studies distinguished between FOG and non-FOG mainly based on the NFOGQ, whereas in our study, this was not the primary method for subgroup classification. To our knowledge this is the first study to use the one-minute 360 turn for classification of freezing subgroups. As a result, many PwPD classified as a freezer had a score 0 on the NFOGQ which influenced this correlation compared to previous studies.

### 4.3 Strengths and limitations

The primary strength of this study is the large multi-centre cohort of 164 PwPD with and without self-reported FOG with variable PD severities. Moreover, FOG was rated according to the current gold standard by two independent raters. This is also the first study to provide cutoff values for each of the FOG-index metrics for classification purposes. However, this study also has several limitations. First, even though this study was prospective, we still had some missing data due to technical problems, having to exclude 13 patients for data analysis. Secondly, sex was not equally distributed between groups. The study cohort had more male freezers than female freezers, which is a typical finding for the PD population at large (4). Third, all measurements were conducted in the ON medication state, so we might have misclassified some unaware dopamine-responsive freezers as non-freezers (16). Further, even though the 360 turn showed to be sensitive for FOG detection, it is very challenging to correctly annotate movements during this task with many short steps and high balance requirements. While the FOG-index offers some insight into FOG classification, it is not yet suitable as a diagnostic tool due to its moderate sensitivity and limited validity. Further development is needed before it can be reliably used in research and the clinic. Future work should also compare the performance of other algorithms (17) to classify PwPD as freezers or non-freezers and assess their criterion validity across various tasks. A limitation inherent to any cutoff-based algorithm, such as the FOG index, is that features in the data need to go beyond a certain cutoff for FOG to be detected. Even though these predefined algorithms are computationally easy to process, they are not personalised to the gait characteristics of each PwPD, nor do they automatically adjust the cutoff depending on the type of gait task performed (26, 34). Even though we found positive results for akinetic FOG using the FOG-index, still many episodes were missed (sensitivity 68.3%). Deep learning methods may be better able to deal with a heterogenous phenomenon such as FOG due to their ability to infer multiple relevant features directly from raw data without the need of manual engineering (35). This makes machine learning a promising technique for FOG classification and quantification (36–38). Lastly, although the DT 360 turn is the most sensitive FOG-provoking task that is quick to perform in a clinical or research setting, this task is a poor representation of daily life ambulation. As a result, the FOG outcome might not fully represent the true impact of FOG on the wellbeing of patients. For safety reasons the turning task was performed under supervision in the laboratory, so we might have missed several unaware freezers due to a white-coat effect. Moreover, as FOG is episodic and unpredictable, repeated testing is indicated for future work.

## 5 Conclusion

The FOG-index (metric AP_Mean) correctly classified freezers from non-freezers with a sensitivity of 68.3% and specificity of 61.7% using a cutoff value of 0.013 when compared against expert ratings of the DT 360 turn. Interestingly, many PwPD were unaware of their FOG yet experienced FOG during this task, indicating that this task might be a sensitive screening tool. Obtaining the FOG-index is more time-efficient than expert classification of FOG, but less precise and should be used with caution. In Addition, low criterion validity was found, meaning the FOG-index is not yet reliable for assessing FOG severity. In conclusion, further development of the algorithm is necessary to improve its classification accuracy and criterion validity for reliable FOG assessment. A future long-term follow-up study is also indicated to assess if the 360 turn can predict future conversion to an aware freezer status.

## Data Availability

The dataset for this study is available upon reasonable request and provision of ethical approval for re-use of the data and data sharing agreement.

## References

[1] Nutt JG Bloem BR Giladi N Hallett M Horak FB Nieuwboer A. Freezing of gait: moving forward on a mysterious clinical phenomenon. Lancet Neurol. (2011) 10:734–44. doi: 10.1016/S1474-4422(11)70143-0, PMID: 21777828 PMC7293393

[2] Schaafsma JD Balash Y Gurevich T Bartels AL Hausdorff JM Giladi N. Characterization of freezing of gait subtypes and the response of each to levodopa in Parkinson's disease. Euro J Neurol. (2003) 10:391–8. doi: 10.1046/j.1468-1331.2003.00611.x, PMID: 12823491

[3] Walton CC Shine JM Hall JM O’Callaghan C Mowszowski L Gilat M. The major impact of freezing of gait on quality of life in Parkinson’s disease. J Neurol. (2015) 262:108–15. doi: 10.1007/s00415-014-7524-3, PMID: 25319020

[4] Kalia LV Lang AE. Parkinson’s disease. Lancet. (2015) 386:896–912. doi: 10.1016/S0140-6736(14)61393-3 25904081

[5] Zhang WS Gao C Tan YY Di CS. Prevalence of freezing of gait in Parkinson’s disease: a systematic review and meta-analysis. J Neurol. (2021) 268:4138–50. doi: 10.1007/s00415-021-10685-5, PMID: 34236501

[6] Gilat M Silva L de Lima A Bloem BR Shine JM Nonnekes J. Freezing of gait: promising avenues for future treatment. Parkinsonism Relat Disord. (2018) 52:7–16. doi: 10.1016/j.parkreldis.2018.03.009, PMID: 29550375

[7] Cui CK Lewis SJG. Future therapeutic strategies for freezing of gait in Parkinson’s disease. Front Hum Neurosci. (2021) 15:741918. doi: 10.3389/fnhum.2021.741918, PMID: 34795568 PMC8592896

[8] Hallett M. The intrinsic and extrinsic aspects of freezing of gait. Mov Disord. (2008) 23:S439–43. doi: 10.1002/mds.21836, PMID: 18668625 PMC4758454

[9] Mancini M Bloem BR Horak FB Lewis SJG Nieuwboer A Nonnekes J. Clinical and methodological challenges for assessing freezing of gait: Future perspectives. Move Dis. (2019) 34:783–90. doi: 10.1002/mds.27709 PMC7105152 31046191

[10] Nieuwboer A Rochester L Herman T Vandenberghe W Emil GE Thomaes T. Reliability of the new freezing of gait questionnaire: agreement between patients with Parkinson’s disease and their carers. Gait Posture. (2009) 30:459–63. doi: 10.1016/j.gaitpost.2009.07.108, PMID: 19660949

[11] Hulzinga F Nieuwboer A Dijkstra BW Mancini M Strouwen C Bloem BR. The new freezing of gait questionnaire: unsuitable as an outcome in clinical trials? Mov Disord Clin Pract. (2020) 7:199–205. doi: 10.1002/mdc3.12893, PMID: 32071940 PMC7011794

[12] Lewis S Factor S Giladi N Nieuwboer A Nutt J Hallett M. Stepping up to meet the challenge of freezing of gait in Parkinson’s disease. Transl Neurodegen. (2022) 11:23. doi: 10.1186/s40035-022-00298-x, PMID: 35490252 PMC9057060

[13] Denk D Herman T Zoetewei D Ginis P Brozgol M Thumm PC. Daily-living freezing of gait as quantified using wearables in people with Parkinson disease: comparison to self-report and provocation tests. Phys Ther. (2022) 102. doi: 10.1093/ptj/pzac129, PMID: 36179090 PMC10071496

[14] Barthel C Mallia E Debû B Bloem BR Ferraye MU. The practicalities of assessing freezing of gait. J Parkinson Dis. (2016) 6:667–74. doi: 10.3233/JPD-160927, PMID: 27662331 PMC5088401

[15] Gilat M. How to annotate freezing of gait from video: a standardized method using open-source software. J Parkinsons Dis. (2019) 9:821–4. doi: 10.3233/JPD-191700, PMID: 31524181

[16] Lewis SJG Factor SA Giladi N Hallett M Nieuwboer A Nutt JG. Addressing the challenges of clinical research for freezing of gait in Parkinson’s disease. Mov Disord. (2021) 37:264–7. doi: 10.1002/mds.28837, PMID: 34939228 PMC8840955

[17] Pardoel S Kofman J Nantel J Lemaire ED. Wearable-sensor-based detection and prediction of freezing of gait in parkinson’s disease: A review. Sensors (Switzerland). (2019) 19:5141. doi: 10.3390/s19235141, PMID: 31771246 PMC6928783

[18] Snijders AH Haaxma CA Hagen YJ Munneke M Bloem BR. Freezer or non-freezer: clinical assessment of freezing of gait. Parkinsonism Relat Disord. (2012) 18:149–54. doi: 10.1016/j.parkreldis.2011.09.006 21968033

[19] D’Cruz N Seuthe J De Somer C Hulzinga F Ginis P Schlenstedt C. Dual task turning in place: a reliable, valid, and responsive outcome measure of freezing of gait. Mov Disord. (2022) 37:269–78. doi: 10.1002/mds.28887, PMID: 34939224

[20] Zoetewei D Ginis P Goris M Gilat M Herman T Brozgol M. Which gait tasks produce reliable outcome measures of freezing of gait in Parkinson’s disease? J Parkinsons Dis. (2024) 14:1163–74. doi: 10.3233/JPD-240134, PMID: 39121137 PMC11380302

[21] Morris TR Cho C Dilda V Shine JM Naismith SL Lewis SJG. A comparison of clinical and objective measures of freezing of gait in Parkinson’s disease. Parkinsonism Relat Disord. (2012) 18:572–7. doi: 10.1016/j.parkreldis.2012.03.001, PMID: 22445248

[22] Mancini M Smulders K Cohen RG Horak FB Giladi N Nutt JG. The clinical significance of freezing while turning in Parkinson’s disease. Neuroscience. (2017) 343:222–8. doi: 10.1016/j.neuroscience.2016.11.045, PMID: 27956066 PMC5289743

[23] Mancini M Hasegawa N Peterson DS Horak FB Nutt JG. Digital measures of freezing of gait across the spectrum of normal, non-freezers, possible freezers and definite freezers. J Neurol. (2023) 270:4309–17. doi: 10.1007/s00415-023-11773-4, PMID: 37208526

[24] Mikolaizak AS Rochester L Maetzler W Sharrack B Demeyer H Mazzà C. Connecting real-world digital mobility assessment to clinical outcomes for regulatory and clinical endorsement–the mobilise-D study protocol. Phillips T, editor. PLoS One. (2022) 17:e0269615. doi: 10.1371/journal.pone.0269615, PMID: 36201476 PMC9536536

[25] Postuma RB Berg D Stern M Poewe W Olanow CW Oertel W. MDS clinical diagnostic criteria for Parkinson's disease. Move Dis. (2015) 30:1591–601. doi: 10.1002/mds.26424, PMID: 26474316

[26] Huang T Li M Huang J. Recent trends in wearable device used to detect freezing of gait and falls in people with Parkinson’s disease: a systematic review. Front Aging Neurosci. (2023) 15:1119956. doi: 10.3389/fnagi.2023.1119956, PMID: 36875701 PMC9975590

[27] Gavriliuc O Paschen S Andrusca A Helmers AK Schlenstedt C Deuschl G. Clinical patterns of gait freezing in Parkinson’s disease and their response to interventions: an observer-blinded study. Parkinsonism Relat Disord. (2020) 80:175–80. doi: 10.1016/j.parkreldis.2020.09.043, PMID: 33027712

[28] McNeely ME Earhart GM. The effects of medication on turning in people with Parkinson disease with and without freezing of gait. J Parkinsons Dis. (2011) 1:259–70. doi: 10.3233/JPD-2011-11030, PMID: 23939306 PMC3912574

[29] Mancini M Shah VV Stuart S Curtze C Horak FB Safarpour D. Measuring freezing of gait during daily-life: an open-source, wearable sensors approach. J Neuroeng Rehabil. (2021) 18:1. doi: 10.1186/s12984-020-00774-3, PMID: 33397401 PMC7784003

[30] Lewis SJG Shine JM. The next step. Neuroscientist. (2016) 22:72–82. doi: 10.1177/1073858414559101, PMID: 25398230

[31] Plotnik M Giladi N Balash Y Peretz C Hausdorff JM. Is freezing of gait in Parkinson’s disease related to asymmetric motor function? Ann Neurol. (2005) 57:656–63. doi: 10.1002/ana.20452, PMID: 15852404

[32] Fasano A Canning CG Hausdorff JM Lord S Rochester L. Falls in Parkinson’s disease: A complex and evolving picture. Move Dis. (2017) 32:1524–36. doi: 10.1002/mds.27195 29067726

[33] Haertner L Elshehabi M Zaunbrecher L Pham MH Maetzler C van Uem JMT. Effect of fear of falling on turning performance in Parkinson’s disease in the lab and at home. Front Aging Neurosci. (2018) 10:78. doi: 10.3389/fnagi.2018.00078, PMID: 29636676 PMC5880950

[34] Zach H Janssen AM Snijders AH Delval A Ferraye MU Auff E. Identifying freezing of gait in Parkinson’s disease during freezing provoking tasks using waist-mounted accelerometry. Parkinsonism Relat Disord. (2015) 21:1362–6. doi: 10.1016/j.parkreldis.2015.09.051, PMID: 26454703

[35] Sarker IH. Deep learning: A comprehensive overview on techniques, taxonomy, applications and research directions. SN Comput Sci. (2021) 2:420. doi: 10.1007/s42979-021-00815-1, PMID: 34426802 PMC8372231

[36] Yang PK Filtjens B Ginis P Goris M Nieuwboer A Gilat M. Freezing of gait assessment with inertial measurement units and deep learning: effect of tasks, medication states, and stops. J Neuroeng Rehabil. (2024) 21:24. doi: 10.1186/s12984-024-01320-1, PMID: 38350964 PMC10865632

[37] Filtjens B Nieuwboer A D’cruz N Spildooren J Slaets P Vanrumste B. A data-driven approach for detecting gait events during turning in people with Parkinson’s disease and freezing of gait. Gait Posture. (2020) 80:130–6. doi: 10.1016/j.gaitpost.2020.05.026, PMID: 32504940

[38] Filtjens B Ginis P Nieuwboer A Slaets P Vanrumste B. Automated freezing of gait assessment with marker-based motion capture and multi-stage spatial-temporal graph convolutional neural networks. J Neuroeng Rehabil. (2022) 19:48. doi: 10.1186/s12984-022-01025-3, PMID: 35597950 PMC9124420

